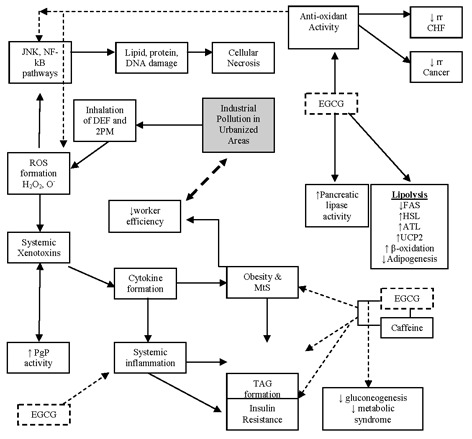# The anti-obesity effects of EGCG in relation to oxidative stress and air-pollution in China

**DOI:** 10.1007/s13659-013-0060-5

**Published:** 2013-10-23

**Authors:** Simon Cichello, Pingsheng Liu, Markendya Jois

**Affiliations:** 1 School of Life Sciences, La Trobe University, Victoria, 3086 Australia; 2 National Laboratory of Biomacromolecules, Institute of Biophysics, Chinese Academy of Sciences, Beijing, 100101 China; 3 Ministry of Education Key Laboratory of Pu’er Tea, Yunnan Agricultural University, Yunnan, 650201 China

**Keywords:** EGCG, obesity, air-pollution, diesel exhaust fumes (DEF), 2PM, reactive oxygen species (ROS), inflammation

## Abstract

Modern China, similar to most developing nations, has seen a rise in the prevalence of both obesity and diesel exhaust based air pollution. The cause of obesity is multi-factorial encompassing diet, lifestyle and social factors. Also there has been a reduction in the consumption of fruit, vegetables, and traditional medicinal foods such as polyphenol containing green tea. Replacing these, are high fat and carbohydrate based processed foods which are quickly displacing these wholefoods in the diet. This review paper proposes evidence that a potential cause of obesity is also linked to environmental stress stimuli such as air pollutants, particularly diesel exhaust fumes (DEF) of > 2.5 μm particulate matter, and discusses a role for a green tea catechin (EGCG) for use as a dietary defence against diet and environmentally induced obesity. China is now at a critical point of a public health pandemic with rising air-borne pollution (via car exhaust fumes DEF), industry pollution such as heavy metals, and the benzene hydrocarbon based ‘2PM’ particulate matter, now accepted as a major environmental issue for public health. Relevant data published in MEDLINE since 1995 has been gathered to formulate the following review.

## 1 Introduction

Obesity is defined as the accumulation of adipose tissue caused by excessive dietary intake of calories or by the reduction of basal metabolic rate (BMR), or in part either in combination that presents a risk to the health of the individual[1]. The factors that influence obesity development, or modulation of BMR, appear numerous and highly complex. Modern China has seen a rise in the prevalence of obesity from as little as 0.2% (1985) to 8.1% (2013), and is affecting males at a higher rate than females[2]. Although dietary patterns have changed, one factor sets China apart; *the business of pollution*. Pollution is being out-sourced to China by the West to mitigate tougher environmental laws and social unrest at a cost that is both seen and unseen, and literally 'felt' in China. Although industry is economically important, the people who drive the systems or live in proximity to industry are adversely affected, possibly impacting productivity. Pollution, in particular $CO_2$ emissions, similar to wealth per capita, has increased exponentially in China over the past 25 years, has now been linked to increased systemic inflammation as documented in a study in mice post-2008 Olympic games[3]. Further, a study of diesel fuel exhaust (DFE) affected maternal mice showed more obese male progeny[4], linking DFE with inflammation and development of obesity.

Traditionally herbs and food were seen as both disease preventative and pro-active medicinal agents in China, such as the use of *Radix notoginseng* for improving lipid profile[5]. However, with rising affluence, increased pollution, poor dietary choices through eating out, and sedentary behaviour, China can now '*have its cake and eat it too*' with unseen association and costs impacting community health and negating improved productivity. With worsening dietary habits in China, particularly in its youth, obesity in 12 to 18-year-olds boy has increased from 7.5%–12.5% from 2004 to 2009[6], appearing as one main influence for the development of obesity. Similar to this, there are a number of phytochemicals in food such as green tea (*Camellia sinensis*) studied, in particular the catechin EGCG which has been observed to display the most effective obesity protective effects, and yet despite this green tea's consumption per capita is decreasing in China. It is literally a '*green paradox*'. Green tea displays a multitude of health benefits, including a pronounced obesity protective role, which is linked in part to the presence of the phytochemical EGCG[7]. This review examines an established function of EGCG in a

*To whom correspondence should be addressed. E-mail: sacichello@hotmail.com

*new light*; application in a seemingly unrelated two-facet public health issue.

Moreover, with the advancement of food technology and nutrition sciences in the 20th and 21st centuries, escalating time pressures due to urbanization has disenfranchised the largely agricultural labour workers of the Chinese populous of their traditional means of food production in peasant/community farms. Instead processed and ready to eat fast foods, as well as distribution centres in urban areas have largely displaced wholefoods such as fruit, vegetables, and herbs in the diet. In a parallel manner, there has been an increase in the prevalence of co-morbidities of obesity such as cardiovascular disease, as well as rise in the proximity of the populous to areas of diminished air quality. The obesity and diabetes protective effects of vegetables may be ascribed to their fiber content, reducing glycaemic load, or the phytochemicals and micro-nutrients such as folate[8], and possibly EGCG in green tea, however how the body reacts to environmental stress stimuli, in particular air and water pollution, may open a new paradigm on obesity and China's economic development and health policies. Both a shift away from these natural dietary sources as well as the compounding effect of environmental stimulants that induce systemic inflammation, are key influences towards the development of obesity in a growing middle-class, urbanized China.

As previously mentioned, a major factor influencing obesity development not widely explored is the role of airborne pollution on fat metabolism. Similar to most developing nations, China has seen an increase in environmental pollution, particularly airborne particles of < 2.5 μm in diameter, in cities such as Beijing, which have been documented to cause the reduction of pulmonary function (PF)[9]. A reduction in PF would reduce an individual's ability for cardiovascular function, exercise, aerobic respiration, and cognitive function and directly influence body composition, and thus work capabilities. A reduction in aerobic respiration is linked to the development of obesity, and the individual's inability to utilize fatty acids by the mitochondria skeletal muscle (SKM) decreases in efficiency and SKM as a percentage of body weight decrease[10], further exacerbating obesity. Compounding this situation further is despite tea production in China increasing, its *per capita* tea consumption continues to decline. Thus, the anti-obesity and anti- inflammatory effects of consuming green tea may be exported to the West, whilst pollution is imported into China. Interestingly, air-pollution, in particular < 2.5 μm particulate matter has been linked to the development of obesity and inflammation in mice[11]. However, the link between dietary intake of black, red, Puer, or, green tea, either as a beverage, food and also medicinal agents and their relation to fat metabolism and possible therapeutic role with pollution induced obesity, remains to be further investigated. This review aims to propose this hypothesis with the support of *in-vitro*, animal and clinical trials.

In addition to diet, lifestyle factors such as reduction in energy expenditure, disintegration of the family home, and occupational stress all contribute to modified behaviour, higher stress and a link with uncontrollable and compulsive eating behaviour which ultimately results in obesity[12]. Specifically, the reduction of dietary micronutrients such as secondary plant metabolites with their effect on fat and glucose metabolism in environmental induced obesity needs more research attention.

**1.1 Relationship of Lipid Droplets and Obesity:** Obesity is defined as the accumulation and enlargement of adipocytes, and regionally reflected in increased visceral fat mass. This increased visceral fat mass is also associated with hepatic insulin resistance, type 2 diabetes and liver steatosis, with systemic inflammation a hallmark by the presence of cytokine hormones[13]. Lipid droplets are organelles, which contain a triacylglycerol (TAG) component used for energy storage, but the protein membrane surrounding the lipid contains proteins that act to communicate to the mitochondria for cellular energy metabolism and fuel preferences[14], and thus BMR. Further, EGCG affects lipid droplets size as a secondary outcome to the protection of cells from cell stress such as autophagy and thus reduce obesity indirectly[15]. EGCG presents as a natural phytochemical readily available in the diet of the Chinese populous that can '*immunize*' against systemic inflammation cause by inhalation of excessive air-pollution, subsequent development of obesity, associated metabolic syndrome, and decreased labour force efficiency.

**1.2 Air Pollution and Induced Obesity:** Recently, there has been growing research showing a link between the ingestion of air-pollution and the development of obesity. In a study of mature C57BL/CBA male mice, the researchers showed that inhalation of heavy industry pollution (steel mills) for a several days, especially the inhalation of < 2 μm particulate matter adsorbed with polycyclic aromatic hydrocarbons, stimulated LD formation in the lungs, possibly acting as a pollutant storage or cell signaller to induce reactive oxygen species (ROS) production, and in-turn endogenous antioxidant mechanisms[16]. Furthermore, prenatal DEF air pollution exposure in mice caused foetal brain cytokine response (systemic inflammation) and predisposed the male offspring to obesity. They also displayed increased anxiety, higher insulin levels (males only) and increased cerebral anti-oxidant response via microglial activation displayed reduced physical activity (males) than their filtered air (FA) counter-parts when they reached adulthood. Furthermore, in a longitudinal cohort study of mothers who gave birth to children born in certain New York boroughs between 1998–2006, revealed that those who experienced higher polycyclic aromatic hydrocarbon (PAH) exposure, pre-disposed the children to a significantly higher body mass, and body fat for obesity at age 5 years, with a relative risk for obesity of 2.26 at the age of 7 years[17]. Moreover, Into et al.[18], observed a relationship between a higher risk factor of child obesity and their respective maternal smoking habits during pregnancy.

Thus, air-pollution, in the form of DE or cigarette exposure during pregnancy in mice or humans has shown to cause both

LD formation in response to increased systemic inflammation, but also adipocyte accumulation and obesity in their progeny. These findings are a major public health concern, particularly in countries where there is a high rate of DE based air pollution, and the involuntary inhalation of this pollution.

**1.3 China, Air Pollution and Inflammation:** Beijing is commonly noted as one of the most polluted capital cities in Asia, and perhaps one of the top 10 most polluted capital cities in the world. Ambient fine particulate matter (diameters < 2.5 μm; PM < 2.5) appears to be the main pollutant from car exhaust as well as industry. Increased air PM < 2.5 is linked to increased morbidity and mortality of cardiovascular diseases. This was evident in male C57BL/6 mice exposed to the Beijing PM < 2.5 air, two months following the Olympics. The mice displayed significant elevation of circulating monocyte chemo-attractant protein 1 in the lung and visceral fat of the mice. As mentioned earlier, this observation may be linked to the elevation of inflammatory cytokine interleukin 6 when compared with filtered air control mice. Phytochemicals in medicinal food and herbal medicine such as catechins in tea may prove to be effective to inhibit certain pathways related to cellular pollutant excretion and systemic inflammation, and be a useful dietary protectant to assist the Chinese populous to protect against systemic inflammation, obesity and pre-mature death due to cardiovascular disease.

**1.4 Phytochemical Relationship with Xenotoxin Excretion:** Phytochemical have been shown to modify the excretion of pollutants such as DEF via multi-drug resistant transports such as p-glycoprotein (PgP)[19]. These toxins, pharmaceutical drugs and some phytochemicals from herbal extracts, are known as *xenobiotics*, and are metabolised via a specialized enzymatic system known as the phase enzymes. For example, the tracheal injection of DE particles such as quinoid or nitroaromatic structures, increases DNA damage via up-regulation of NADPH-cytochrome P450 reductase. Conversely, this up-regulation was reduced by endogens superoxide dismutase (SOD), and catalase enzyme activity[20]. Further, glutathione S-transferases are key enzymes which are important in the detoxification process, and are able to transport conjugated toxins following phase II processing outside of the cells[21]. The phenolic metabolites of benzene such as hydroquinone, catechol and 1,2,4-benzenetriol, may accumulate in fat tissue and subsequently be released upon ingestion of a lipolytic product such as EGCG in green tea. The direct elimination from cells of conjugated toxins is usually via the trans-membrane PgP[22], with the elimination via the urine, bile etc., after processing. However, green tea does not seem to be responsible for the direct modulation of cytochrome P450 enzymes[23], but may instead modulate the body composition in response to these xenotoxins, acting as a phytochemical protective mechanism against air-pollution induced obesity, chiefly ascribed to its anti-oxidant properties.

## 2 Phytochemicals in Green Tea Responsible for Body Composition Modulating Effects - Catechins & Caffeine

Green tea possesses a number of phytochemicals (see Table 1 and Figures 1 & 2). One of those chemical groups is the catechins, or flavan-3-ol (flavanol), a secondary metabolite of the tea plant. The catechins commonly found in green tea include epigallocatechin (EGC), epicatechin (EC), epigallocatechin gallate (EGCG), and epicatechin gallate (ECG)[24]. Green tea also contains the methylxanthine, caffeine.

**Figure 1.** EGCG, a catechin found in green tea (*Camellia sinensis*)

**Figure 2.** Caffeine

## 3 Overview of Green Tea

As shown in Table 1, the main phytochemicals present in green tea include hydrolysable tannins such as EGCG and also the methylxanthine caffeine. The polyphenol content of green tea has been suggested to reduce ROS such as reduction in lipid peroxidation in metabolic disorders such as obesity[26].

**3.1 ROS Formation and Role of Anti-Oxidants:** Reactive oxygen species are chemicals that contain oxygen, but are highly reactive oxygen such as $O^-$ and $H_2O_2$, natural by-products of metabolism. Stress via dietary or environment influences can increase ROS production, leading to 'oxidative stress'. Oxidative stress can manifest physically in a number of positive and negative ways; positive – apoptosis of undesirous cells (i.e. malignancies), platelets involved in wound repair, blood homeostasis, and in a negative way with ROS implicated in a number of morbid conditions such as CHF, obesity and diabetes. Further, ROS causes DNA damage, oxidation of polyunsaturated fatty acids in the lipid membranes (lipid peroxidation) and the oxidation of amino acids in proteins. Certainly, it has been demonstrated that pre-pubescent children with excessive fat accumulation appear to induce higher levels of ROS, linking it to obesity[27]. SOD and catalyse are endogenous enzymes with anti-oxidant activity that modulate plasma ROS and are also increased in the ingestion of tracheal injected DE particles, as previously mentioned. EGCG is proposed as a phytochemical that protects against ROS formation by the excessive ingestion of air-pollution.

**Table 1. Outline of therapeutic action of green tea and active constituents**

| Common Name (*Latin binomial*) | Part Used / Active Constituent(s) | Main Action & Indication |
|---|---|---|
| Green Tea (*Camellia sinensis*) | Leaf<br>**Hydrolysable tannins:** polyphenol (catechin), epigallocatechin gallate (EGCG); **Methylxanthines:** caffeine | • anti-oxidant, astringent, anti-bacterial, anti-viral, hypo-cholesterolaemic, diuretic, anti-carcinogenic<br>• Indicated in cancer, Alzheimer's disease, cardiovascular disease. Modification of body composition (fat content), adjunct therapy in treatment of cancer, and hyper-cholesterolaemia[25]. |
| Other | Flavanoids, Triterpenoid Saponins, Theanines, Theaflavins | |

Fortunately, the body also has a number of endogens and exogenous defence systems to mitigate the harmful effects of ROS, including anti-oxidants. Anti-oxidants are a group of chemicals that protect against the deleterious effects of oxygen radicals. They include SOD, catalase, glutathione peroxidises (endogenous forms), and dietary forms; ascorbic acid, tocopherols and green tea polyphenols. In a study of human lymphocytes challenged with $H_2O_2$, *in vitro* pre-treatment with 0.1% w/v green tea and also a parallel human trial (300 mL/d, 6 weeks) of green tea reduced DNA damage[28]. Ingestion of air-pollution is a major environmental cause of systemic inflammation in urban populations or populations residing within the vicinity of factories in China. Green tea polyphenols may provide the necessary '*biological filtering*' needed to reduce oxidative stress, and associated increased incidence of obesity. Chinese food manufacturing needs to develop novel methods of ingesting green tea catechins in other beverages and food items is necessary in particular EGCG, and retain tea products for local consumption if they want to reduce the health burden of environmental pollution, obesity and associated economic costs of fatigue and reduced productivity *per capita*. The macroeconomic and health epidemiology analogy is to '*sacrifice the golden goose to maintain the farm's up-keep'*. The multitude of morbid conditions such as cancer and cardiovascular disease, have ROS and systemic inflammation as hallmarks of their disease prognosis and are serious concerns for the ageing middle class.

**3.2 Relationship of Air-Borne Pollution and Increased Systemic Inflammation and Proposed Modulation by Green Tea Polyphenols:** In a study by Chuang et al.[29], the inhalation of particles with aerodynamic diameters i.e. < 2.5 µm (2.5 PM), sulphate etc. models, were found to be correlated with increases in inflammation C-reactive protein (CRP), 8-OHdG), fibrogen, PAI-1 (inflammation, oxidation stress and blood coagulation respectively) and autonomic dysfunction simultaneously in healthy young humans, living in highly urbanized areas. Polyphenols found in green tea may present a natural method to control oxidative damage caused by the inhalation of air-borne pollutants and resultant in obesity development such as benzene hydrocarbons or its metabolites. Lowering ROS formation using dietary polyphenols such as EGCG may also decrease the incidence of obesity in urban pollutions subjected to high levels of air-pollution, as a secondary outcome of mitigating systemic inflammation.

Green tea extracts have been noted to modulate ABC family transporter gene expression and protein function such as PgP and multidrug resistance-associated protein 2 (MRP2)[30], and reversing multi-drug resistance in cancer cell lines. It was further revealed that EGCG down-regulated MDR1 gene expression and shows promise in drug resistant cancer treatment[31]. On the other hand, when exposed to DE fumes (DEF), P-glycoprotein, a trans-membrane multi-drug resistance protein is up-regulated in the blood-brain barrier during DEF ingestion. It ameliorates increased plasma TNF-$\alpha$ due to DEF, and reduces the inflammatory response due to oxidative stress. Further, Hartz et al. 2008 showed that pre-treatment using radical scavengers such as EGCG, ameliorated DE-induced PgP up-regulation in capillaries, or the inhibition of NADPH oxidase, via inhibition of JNK and inhibition of cytokine induced apoptosis pathway, but not NF-κB pathway inhibition. Moreover, green tea treatment has been suggested to be protective against oxidative damage caused by xenotoxins[32] e.g. DEF, possibly via ABC transporters such as P-glycoprotein[33].

The relationship of EGCG and PgP *in vivo* may be synergistic for the excretion of harmful air-pollutant toxins and their metabolites such as DEF, cigarette smoke (CS). EGCG has been shown to reduce lung morphological changes such as airspace enlargement and goblet cell hyperplasia; excessive mucus production and also oxidative stress in rats exposed to CS. The proposed mechanism is the suppression of CS-induced oxidative stress[34]. The suppression of oxidative damage may be related to the reduction of systemic inflammation. At a molecular level, green tea polyphenols (GTP) (200 mg/Kg/d) have been shown, in conjunction with a high fructose diet, to reduce the detrimental effects of insulin signalling. Further, this GTP dosage modulates lipid metabolism and reduces inflammation in rat cardiac muscle[35]. The precise mechanisms proposed vary, but EGCG certainly reduces ROS and oxidative stress due to environmental influences such as air-pollution (i.e. DE) as depicted in Figure 3.

**3.3 Proposed Role of Green Tea Dietary Treatment of Oxidative Stress by Air-Borne Pollution Induced Obesity:** Green tea compounds have been observed to display anti-oxidants effects mainly via the up-regulation of the gene Nrf2, a primary cellular anti-oxidant defence against cytotoxic effects of oxidative stress, and protection against $H_2O_2$ induced

**Figure 3.** Diagrammatic summary, representing the hypothesized effect of DEF and 2PM pollution to induce systemic inflammation and obesity. Further, EGCG is shown to reduce obesity (anti-obesity effect) and thus reduce adverse physiological effects of DEF, 2PM pollution. Abbreviations not represented in the text: ↑ increase, ↓ decrease, rr: relative risk, TAG: Triacylglyceride.

apoptosis[36]. Green tea extract polyphenols also increase phase I and phase II enzyme activity in vivo[37]. Therefore, a diet rich in anti-oxidants such as EGCG is essential to reduce oxidant damage via ROS generation in phase I, in addition to consuming glutamic acid, cysteine and glycine containing foods, as liver GSH is an important endogenous anti-oxidant [38]. In addition, theanine is a glutamate derivative found in tea, which has been observed to decrease the induction of the lipid peroxide and glutathione peroxidise. The mechanism was proposed as the inhibition of GSH reduction in the liver and heart[39], with GSH showing a protective effect against benzene toxicity in gasoline pump workers[40]. The proposed mechanism by which green tea polyphenols as a group or specific phyto-chemicals such as EGCG, and theanine, may attenuate the effect by GSH is to protect against DNA damage is by ROS.

The formation of polyphenolic metabolites of benzene such as hydroquinone, catechol, 1,2,4-benzenetriol and pyrogallol, produce various ROS (namely $H_2O_2$, $O_2^-$, HO) causing DNA damage and reduction of GSH. This damage may be ameliorated by dietary consumption of GTP. However, phase II enzymes are elevated by both GTP and phenolic metabolites of benzene, whereas the GTP counterbalances the reduction of GSH, being especially antagonistic to the hydroxyl radical[41]. EGCG appears to effect hepatic detoxification enzymes, but also may be modified *in vivo* by intestinal bacteria, and hepatic conjugation to exert its effects as a conjugate.

**3.4 Absorption and Hepatic Modification of Green Tea via Hepatic Conjugation:** Green tea catechins have been shown to be poorly absorbed in the small intestine, often post-absorption, they are conjugated to glucuronic acid, sulphate groups etc., to leave a polyphenolic skeleton excreted in the urine[42]. Further, the effects of green tea catechins (total 85%) have been shown to influence the enzymatic system cyto-chrome P450 oxidase (CYP) (P450 oxidases conjugates polar groups onto a xenotoxin), by both GTE and EGCG. Interestingly, both GTE and ECGC competitively inhibited CYP2B6 and CYP2C8 variants of CYP, and non-competitive inhibition against CYP3A in human liver microsomes, at concentrations of 5.9, 4.5, 48.7, 25.1 and 13.8 µg/mL respectively. In human intestinal microsomes, GTE and EGCG inhibited CYP3A at 18.4 µg/mL and 31.1 µM, respectively in a non-competitive manner. These results suggest that green tea catechins cause clinically relevant interactions with substrates for CYP2B6 and CYP2C8 in addition to CYP3A[43]. Hepatic inhibition of CYP450 enzymes may be linked with the reduction in systemic inflammation via reduced ROS formation, as toxins such as metabolites of benzene are conjugated in phase II reactions making them less volatile in the cell, and less likely to cause damage. Moreover, GTE has been shown in aflatoxin B1 challenge in pigs to enhanced glutathione S-transferase activity. GSH conjugates AFB1 to glutathione GSH in the intestine. Ultimately, AFB1 detoxifica-tion is facilitated via cytochrome 450 enzyme activity in the liver and the enhancement of GST activity in the intestine[44]. Without the protective effects of GSH, benzene hydrocarbon pollution may continue to cause systemic inflammation and induced obesity in heavily polluted urban and city populations.

**3.5 Green Tea and Anti-Systemic Inflammatory Effect:** Green tea consumption has been associated with a reduction in systemic inflammation. The inflammation inhibition effect of EGCG is well noted by the inhibition of nuclear factor-κB (NF-κB) pathway. NF-κB is a DNA transcription factor of many inflammatory mediators such as $NOS_2$ and thus inhibiting NO production a known facilitator of cellular oxidative stress[45]. In a mouse model of polymicrobial sepsis, intraperitoneally injection of EGCG (10 mg/kg) ameliorated hypotension and increases the survival of mice with sepsis[46]. As previously mentioned, cigarette smoke increases oxidative stress, and inflammatory cellular responses. The dietary ingestion of green tea extract (3 mg/d, or 4.5 mg/d) increased the release of blood high mobility group box-1 (HMGB1) and elevated serum cotinine level, also confirmed in a murine macrophage cell lines[47]. Moreover, EGCG (dosage of 20 and 40 mg/kg) has been observed to reduce systemic inflammation and oxidative stress as it inhibits tumour growth, reduced serum CRP, lipid peroxidation in mice bearing a solid Ehrlich ascites carcinoma[48]. In addition, a study of 24 female LEWIS rats, fed with chow and a 0.1% polyphenolic extracts from green tea were shown to reduce/ inhibit hepatic necrosis, oxidative stress (4-hydroxynonenal), IL-6 systemic cytokine release and apoptosis via caspase-8 and Bax, when rats were haemorrhaged via reduced plasma alanine aminotransferase and lactate dehydrogenase as well as down-regulation of both JNK and NFκB leading to reduced hepatic apoptosis[49].

The reduction in systemic inflammation by GTE in sepsis, tumour growth and hepatic necrosis may be a ubiquitous mechanism by which EGCG may reduce systemic inflammation, thus reduction of obesity by inhibition of adipocyte differentiation, decreased fatty acid synthase and increased lipolysis, and in summation a pronounced anti-obesity effect. The anti-inflammatory effects of EGCG as shown in these outlined disease states may also apply to inflammation cause by excessive ingestion of air-pollution, thus modulating the adverse effects of body composition modulation such as excessive accumulation of adipose tissue.

**3.6 Anti-Obesity and Body Composition Modulating Effect of EGCG:** As previously mentioned, green tea, and more so EGCG and caffeine have well established anti-obesity actions both *in vitro* in cell culture and *in vivo* in animal and human models of obesity. In both animal and human studies, EGCG has been demonstrated to prevent the effects of genetic and diet induced obesity (DIO), insulin resistance and hypertension, via reduced dietary fat absorption and metabolism modulation, increased glucose utilization, decreased *de novo* lipogenesis, and anti-oxidative effects[50]. Both the protective insulin resistance, hypotensive and hypocholesterolemic effects of EGCG are related to its overall anti-obesity effect via fat metabolism modulation.

A mouse (C57BL/6J) and sprague dawley rat models of obesity using TEAVIGO tea extract (primarily EGCG) decreased sub-cutaneous and epididymal adipose tissue weights, plasma glucose, triglycerides, and leptin[51]. However, in a study of women with a BMI > 27 $kg/m^2$ that consumed a supplement comprising of GTE of 400 mg [302 mg EGCG] thrice daily for 12 weeks, resulted in only a minor change of 0.3% reduction in body weight, and respective reductions in LDL-cholesterol and triglyceride, with parallel increase in adiponectin and ghrelin suggesting increased $\beta$-oxidation and suppression of gluconeogenesis[52]. Limitations of the study include the lack of an exercise regime and also body composition analysis such as waist: hip ratio. It may be probable that the women may have increased muscle mass (thus fluid accumulation) and decreased adipose tissue with weight remaining stable. Interestingly, in a mouse model of diet induced obesity, 2% of a dietary sucrose substitution with Japanese green tea in a high sucrose (36.1%), high butter fat (38%) diet, significantly decreased total body fat, fatty liver, liver weight and also increased total muscle mass[53], possibly via the EGCG content of the diet, as also observed by Bose et al.[54] using a 3.2 g/kg of diet dosage in a high fat diet (60% energy as fat). Further, Basu et al. 2010 showed in a randomized human trial of obesity and metabolic syndrome, using a control group (4 cups water/d), or treatment; 4 cups of green/d) (treatment a), or green tea extract (2 capsules of either 400 or 800 mg and 4 cups water/d) (treatment b) observed them for 8 weeks. All were matched for EGCG content except the control group. Both treatments (a) and (b) induced decreased body weight at 8 weeks, decreased LDL-cholesterol, decreased lipid peroxidation enzymes which correlated with plasma EGCG levels. Moreover, in postmenopausal women, supplementation of EGCG as a GTE reduced glucose, insulin and LDL-cholesterol with both 400 mg and 800 mg/d over a two months period. In a study using 0.5% dietary green tea polyphenols in a high-fat diet group for 4 months, was divided into two groups either consuming the GTP in the drinking water or food. Compared with the control high fat (HF) diet group, the GTP supplementation increased lean mass, bone mineral density and strength, and decreased pro-inflammatory cytokines and IGF-1[55]. Furthermore, a decaffeinated green tea extract consisting of 530 mg total, containing 400 mg total catechins/capsule, was administered twice daily and was shown to decrease body weight versus placebo groups suggesting a weight gain protective effect[56]. Direct decreased body weight by green tea catechins such as EGCG, in particular adipose tissue is a direct result of increased lipolysis, and decreased apipogenesis/lipogenesis, which may be a beneficial effect to reduce obesity development via increased systemic inflammation by inhalation of air-pollution and in combination with a rapidly changing diet in urbanized mainland Chinese.

**3.7 EGCG Induced Increased in Lipolysis, Decreased Adipogenesis:** Lipolysis involves the catabolism of lipids into free fatty acids via triglyceride hydrolysis. They are transport in plasma by albumin protein and lipoproteins (VLDL, LDL, HDL) respectively. The next step known as $\beta$-oxidation degrades free fatty acids into acetyl-Co A which then enters the TCA cycle.

In a two month dietary intervention of a human DIO trial, Yang et al.[57] observed that daily consumption of a catechin rich tea (650 mL), reduced body weight by up to 20%, via increased lipolysis. WAT loss, specifically mediates a number of genes involved in adipogenesis, lipolysis, $\beta$-oxidation and thermogenesis in white adipose tissue (WAT)[58]. EGCG has been observed to also down-regulate adipogenesis genes such as peroxisome proliferator-activated receptor (PPAR)γ, fatty acid binding protein ((FABP)4), fatty acid synthase (FAS),

LPL, adiponectin etc[59]. In a male C57BL/6J mouse model fed a high-fat diet for 8 weeks induced obesity were then provided with dietary supplementation of 0.2 or 0.5% EGCG (w/w) for a further 8 weeks. This resulted in a reduction of body weight, adipose tissue mass, decreased plasma triglyceride and liver lipid. When examined for adipogenic genes the epididymal WAT the EGCG diet-fed mice displayed reduced adipogenic gene mRNA levels of PPAR-γ, LPL and FAS were significantly decreased. However, mRNA levels of carnitine palmitoyl transferase-1, uncoupling protein (UCP 2), hormone sensitive lipase (HSL) and adipose triglyceride lipase, were significantly increased. These results suggest that EGCG acts to increase energy metabolism, and lipolysis as mechanisms to decrease WAT in addition to its anti-inflammatory effect.

As mentioned, ECGC decreases fatty acid synthesis. Fatty acid synthesis occurs via substrates such as acetyl-CoA and malonyl-CoA and the enzyme fatty acid synthases. In a study of MCF-7 breast cancer cells, Lin et al.[60] observed the hypo-lipidemic and anti-obesity effects of various teas including green tea are due to the inhibition of lipogenesis, in part due to down-regulated fatty acid synthase (FAS) mRNA expression, and up-regulation of energy expenditure in the mitochondria via protein kinase B)/Sp-1 signal transduction pathways (apoptosis pathway). Furthermore, another study examined the polyphenol effect from green tea, and gomchui tea (*Ligularia fischeri*) that all exhibit anti-obesity effects via inhibiting pan-creatic lipase, in the following order; gomchui tea > coffee > green tea. EGCG was the most potent polyphenol to inhibit lipases however the di-O-caffeoylquinic acid isomers (DCQA) from gomchui tea were observed to be more stable during digestion. Thus, they displayed stronger lipase inhibitory activity during simulated digestion rather than EGCG[61].

Moreover, EGCG (20μM) has been observed to reduce Glycerol-3-Phosphate Dehydrogenase (GPDH) activity by 50%, whereas other catechins inhibit GPDH at a concentration of > 100 uM. GPDH catalyzes the *β*-nicotinamide adenine dinucleotide (NADH)-dependent reduction of dihydroxyace-tone phosphate (DHAP) to yield glycerol-3-phosphate, which serves as one of the major precursors of triacylglycerols[62]. Therefore, EGCG directly affects enzyme kinetics in fat synthesis directly effecting formation of the precursor mole-cules. Both fatty acid and glucose metabolism dysfunction manifest as metabolic syndrome, with EGCG possibly being effective to reduce the severity of metabolic syndrome. Further, although lipase inhibitory is up-regulated by EGCG, fat mal-absorption would explain part of its effect on modulating fat metabolism, and it may appear elusive where the metabolic energy of fat oxidation is being used, with non-digestive or non-shivering thermogenesis proving to be a likely candidate.

Moreover, EGCG has been shown to decrease or even inhibit adipocyte differentiation. In a high fat diet model of obesity, TEAVIGO tea extract (primarily EGCG) was fed to mice (C57BL/6J) and Sprague-Dawley rats which resulted in decreased body weight (subcutaneous and epididymal adipose tissue weights), plasma glucose, triglycerides, and leptin, conferring with the inhibition of adipocyte differentiation *in vitro,* as evidenced by Wolfram et al. 2005. Furthermore, at a concentration of 10 μM, EGCG entirely inhibits adipocyte differentiation as evidenced in a mouse embryonic fibroblast-adipose like cell line (3T3-L1)[63]. This observation may be only one mechanism by which EGCG ameliorates fatty liver via inhibition of adipocyte differentiation, and increased lipolysis.

EGCG modulates fat metabolism by various mechanisms including malabsorption, increased lipolysis, decreased synthesis and adipocyte differentiation inhibition. Specifically, increased fatty acid lipolysis, requires further discussions to clarify key mechanisms to explain the apparent weight and fat loss, but may entail increased fatty acid oxidation and mitochondrial protein regulation. Amelioration of fatty body composition induced by PM2 systemic inflammation is required to address the current health epidemic.

**3.8 EGCG Effect on Fatty Acid Oxidation and Thermogenesis:** Fat oxidation or *β*-oxidation entails the degradation of fatty acids into acetyl-Co A sub-units which join the TCA cycle to produce ATP. *β*-oxidation occurs in the mitochondria and is increased in moderate exercise. Exercise maintained at 60% of maximal oxygen consumption ($VO_2$max), with the consumption of 890 ± 13 mg green tea polyphenols, with 366 ±5 mg EGCG, versus a placebo diet of corn-flour (1729 ± 22 mg), increased fat oxidation rates by 17% and in turn the proportion of energy expenditure due to fat oxidation increased in parallel with EGCG dosage[64]. Further, in a double blind placebo study EGCG supplementa-tion with and without caffeine consumed for 3 days, increased fat oxidation within < 2hrs of a meal (+ 34.5%) and (+ 49.4%) respectively, and > 2hrs to increase postprandial fat oxidation (+ 26.3%) and (+ 35.4%) for the caffeine and EGCG/caffeine groups respectively. ECGC effects energy expenditure in combination with EGCG[65]. Further, in high fat-fed C57bl/6J mice treated with 0.32% dietary EGCG for 16 weeks, the mice displayed reduced body weight gain and final body weight (19.2% and 9.4% respectively) compared to high fat-fed controls, possibly via reduced insulin resistance by 33.9%. Further, EGCG reduced fatty liver, with increased skeletal muscle UCP3, and PPAR-*α* by 1.4-1.9-fold compared to high fat-fed controls. These genes are all related to mitochondrial fatty acid oxidation, with the mice also had increased faecal lipid content of faeces[66], suggesting both fat malabsorption and increased fat oxidation. Fat oxidation may also concurrently work synergistically with the down-regulation of fatty acid synthesis enzymes in the liver and adipose tissue, with a cer-tain percentage of fat malabsorbed and thus potential energy intake by-passed as the faeces.

In a three month intervention trial of human DIO model of moderate obesity[67] observed that green tea extract AR25 (25% catechins as EGCG), decreased body weight via increased thermogenesis. Also another proposed mechanism was the inhibition of gastric and pancreatic lipases, however this observation was not shared by Boschmann et al.[68] who provided a 300 mg of EGCG/d for a short 2 d period. However, the limitations of this study were the small sample size (n = 6) and a short study duration of only 2 days, however other environmental factors such as temperature may also be involved in green tea (effect of EGCG and caffeine) induced thermogenesis. Moreover, during cold exposure (3hr exposure time), 1600 mg of EGCG and 600 mg of caffeine have been shown to effect non-shivering thermogenesis, reducing the shivering intensity by 20% and increase total AUC for energy expenditure by 10%[69]. It would appear the cycle of lipolysis and thermogenesis to be a steady state, but the physiological mechanisms governing EGCG effect on adipose tissue also may favour the concurrent inhibition of adipocyte differentiation.

Green tea has been observed to be beneficial for general obesity through a variety of mechanisms, but the reduction of inflammation, lipolysis, and inhibition of fatty acid synthesis and also adipocyte differentiation, seem to be likely candidates (see Figure 3). Results have emerged showing that EGCG and the EGCG free fractions regulate the gene expression of lipid metabolism enzymes, particularly the suppression of the lipogenic enzymes such as FAS, hydroxymethylglutaryl coenzyme A reductase, and acetyl-coenzyme A carboxylase $\alpha$[70]. It is probable that this result was observed through other catechins and also the effect of caffeine in the non-EGCG water soluble fraction. Other lifestyle factors such as exercise may also potentiate both reduction in metabolic syndrome symptoms, reduced weight loss, increased lipolysis and hypoglycaemia in part or combination with dietary consumption of EGCG as a whole supplement or as a tea to ameliorate obesity.

**3.9 Exercise in Combination with EGCG Intake:** Aerobic endurance exercise has been observed to increase fat utilization, improve cardiovascular risk via reduced plasma cholesterol and improved glucose metabolism efficiency as evidenced by reduced HbA1c in type II patients[71]. Furthermore, the ingestion of a EGCG supplement (TEAVIGO) (150 mg twice daily) combined with exercise in overweight/obese postmenopausal women exercised at moderate intensity for 12 weeks caused the reduction in total body fat, specifically reduction in waist circumference via reduction of abdominal fat, and intra-abdominal adipose tissue, possibly via a hypoglycaemic effect[72]. Furthermore, Venables et al. 2008 observed in healthy young men, that GTE ingestion (acute intake) can increase fat oxidation during a moderate exercise regime and is connected with glucose metabolism as insulin sensitivity is significantly improved. Moreover, in a 10 week double blinded placebo control) exercise program study, the ingestion of a caffeine-EGCG-containing drink prior to exercise in overweight and obese women, significantly increased the muscle mass (MM), decreased total cholesterol with a significant time × training interaction for peak oxygen consumption observed. Further, there was a significant improvement in upper-body and lower-body strength ($p < 0.05$), thus improving MM, fitness, and lipid profiles in overweight women[73]. Reduced pulmonary and cardiovascular function due to inhalation excessive air-pollution may further compound an urbanized person's ability to exercise, leading to accumulation of not only air-pollution toxins discussed but also but further development of fat accumulation, inflammation, and creation of a cyclic event that debilitates the individual in a perpetual state of oxidative stress. Another factor not suggested yet is appetite, one key variable affecting calorie intake with EGCG possibly causing satiety.

Interestingly, in a study using a dietary supplement containing 170 mg of N-oleyl-phosphatidylethanolamine (NOPE) and 100mg of EGCG were shown to improve patient compliance to low calorie diet during regular, and moderate exercise (for four weeks), but then failed thereafter for an additional 4 weeks. Subjects reported experiencing fatigue and confusion however there was significant differences for body mass or composition[74]. Although fat metabolism appears to be the key area of body weight modulation, the shift in lipid utilization may be influenced by glucose metabolism.

**3.10 ECGC Effect on Glucose Metabolism:** EGCG has been shown to influence glucose metabolism. In a two months trial administering 544 mg polyphenols and 456 mg as green tea catechins with the consumption of tea as a beverage, amounting to a total daily polyphenol content from tea and supplement amounting to 747 mg/day versus the 469 mg in the control group. There was a non-significant reduction in the body weight, and also BMI related to reduced plasma glucose and Hb A1c (glycated haemoglobin levels), whereas the reduction in insulin level was related to polyphenol content and HOMA index ($\beta$-cell function and insulin sensitivity index incorporating circulating pro-insulin)[75], and in combination with exercise, EGCG intake for 12 weeks in exercising obese woman significantly reduced plasma glucose and also impaired GTT. EGCG on its own, possibly with the influence of caffeine, may induce hypoglycaemia, with a potentiated effect of exercise. Caffiene has been shown to be one of the main hypoglyceamic compounds in green tea, but appears to also impair insulin sensitivity[76].

Green tea polyphenols (GTE), in a study by Venables et al. 2008, showed that healthy men consuming either a 890 ± 13 mg of polyphenols and 366 ± 5 mg of EGCG per day or a placebo (corn-flour; 1729 ± 22 mg), provided for 24hrs prior to performing exercise (cycling 30 minutes; maintaining a 60% of maximal oxygen consumption ($VO_2$max), decreased insulin AUC with a parallel increase of 13% in insulin sensitivity. On the contrary, the supplementation of 400 mg capsules of EGCG twice daily in middle aged overweight and obese males subjects for an 8 week period revealed no effect on insulin sensitivity, secretion or glucose tolerance. The only result was a modest reduction in diastolic blood pressure and some improvement in mood[77]. This is further supported by Hsu et al. 2011[78], who reported green tea extract (856 mg of EGCG) did not affect anthropometric measurements, fasting glucose, haemoglobin A1C percentage (HbA1C), hormone peptides, or plasma lipoproteins in obese individuals with type 2 diabetes. It appears that EGCG may affect insulin sensitivity in healthy males rather than type II diabetic middle aged males instead. The possible mechanism for insulin inhibition is via the effect of EGCG on adipose tissue, and to the pancreas via a secondary hormonal signaller. It was observed that the action of insulin was inhibited (by 50%) by EGCG directly (5–10 μM) in 3T3-L1 and C3H10T1/2 adipocyte cell lines. Insulin stimulation of adipocytes was inhibited by EGCG in a dose/ time-dependent manner (2 h), thus inducing apoptosis, with other catechins/ phytochemicals effective in decreasing order; EGCG > EC> EGC > ECG. Adipocyte pre-treatment with the 67LR antibody (67LR is an extracellular matrix glycoprotein, often up-regulated during certain forms of cancer), reversed the inhibition of insulin by EGCG, and thus the 67LR receptor may be one modulator of EGCG inhibited glucose absorption in adipogenesis[79]. Hseih et al.[80] documented that EGCG exerts anti-insulin action via adipocyte glucose uptake via the AMPK, and that EGCG mediates lipolysis to render TAG available for $\beta$-oxidation. Further, EGCG down-regulates gluconeogenic enzymes such as glucose-6-phosphatase, phosphoenolpyruvate carboxykinase in mouse hepatocytes)[81]. Further, modulation of glucose and lipid metabolism by green tea, may also be in part due to modulation of adiponectin[82], a hormone that increases β-oxidation and also suppresses gluconeogenesis. Hormones such as leptin, insulin and adiponectin may be pivotal to metabolic syndrome, glucose and fat metabolism dysfunction in obesity, with EGCG being a dietary supplement that has been shown to modulate the symptoms of metabolic syndrome.

**3.11 EGCG Effect on Metabolic Syndrome:** Metabolic syndrome (MtS), represents an array of symptoms ranging from increased blood pressure, hyperglycaemia, excess visceral body fat, and fatigue. EGCG has been observed to ameliorate MtS. The male mice (C57BL/6J) were fed a high fat (HFW; 60% as fat) diet, and compared with the HF group, the HFW group gain more body weight and also developed increase severity of metabolic syndrome (i.e. decreased BMR, fat accumulation and hypoglycaemia). Bose et al. 2008 proposed that EGCG treatment protects against both fat accumulation in body weight via increased faecal lipids, lower hepatic lipids, plasma cholesterol and hypoglycaemia, reduced insulin resistance and pro-inflammatory molecules such as CRP, IL-6, and granulocyte colony-stimulating factor (G-CSF).

Ultimately, the dysfunction of lipid, glucose metabolism, with the development of metabolic syndrome and co-morbidity with CHF and cancer all cause of a shortened life span. EGCG may be one phytochemical that can modulate adipose tissue to improve both the quality of life and life span. Further, metabolic syndrome is associated with the up-regulation of lifespan determinant pathways (i.e. silent information regulator, SIR) T1, p66Shc, and mammalian target of rapamycin (TOR)[83]. It is associated with substrate availability and development of visceral adipose tissue, insulin and IGF-1 levels, all of which have been observed to be modulated by EGCG.

**3.12 Green Tea, EGCG and Longevity – Influence of Obesity and Calorie Intake:** Longevity is rather a subjective term as differs both for genders, different populations within and between countries have different life expectancy that determines a standard lifespan. Green tea has been shown to extend lifespan in lower organisms such as *Caenorhabditis elegans*, in particular the phytochemical theanine (conc. 100 nM)[84]. Although green and black tea have been demonstrated to extend the life span of *Caenorhabditis elegans*, Drosophila and in some mouse models, in a study of male F1 hybrid mice and lifespan, it appeared green tea treatment had no effect. Interesting, in the same trial, a 40% calorically restricted diet induced extended lifespan. The green tea group did reduce body weight and fat, although the authors suggested that the polyphenol content does not contribute to longevity, even though the consumption of whole fruits and vegetables is associated with potentiated health and life span[85]. There may be several factors associated with higher fruit and vegetable consumption, such as lower calorie to nutrient ratio (nutrient dense foods increased fibre), and low calorie content due to the inherent water content (80–90%) which would aid satiety and restrict over-eating. Further, the fibre content of fruit and vegetables has been shown to modulate the absorption of carbohydrates (lower glyceamic index), and thus effect glucose metabolism (i.e. insulin sensitivity), as evidenced in a study examining the intake of a low GI soy based powder cake to suppress postprandial blood glucose but not insulin[86]. Lastly, higher fibre ($\beta$-glucan) lowers LDL cholesterol, a factor in the aetiology of heart disease, and thus a lower risk of a major morbidity factor in longevity, CHF. These effects may reduce morbid disease and improve quality of life leading to a longer life span, even in highly polluted cities. Green tea included in food mould increase polyphenol and fibre content contributing to longevity.

In agreement with these findings, green tea intervention for four months was shown in mice does not extend lifespan *per se*, however may reduce the risk of mid-life deaths in female mice[87]. Interestingly, in a human trial of middle aged Japanese men with hypertension (undergoing treatment) and also mitochondrial DNA 5178 cytosine/adenine (Mt5178C/A) polymorphism (associated with longevity). There was an association between Mt5178C genotype and hypertension which was found to be dependent on green tea consumption (lower odds ratio for hypertension)[88]. However, in mice who received 80mg/l in drinking water of green tea polyphenols displayed longer life spans (+ 6.5%) than their control counterparts[89], possible due to EGCG content specifically. This effect was also seen in wild-type N2 and transgenic strains of *Caenorhabditis elegans*, due to regulation of intracellular levels of $H_2O_2$, which were inhibited by 220 μM EGCG, which extended the mean lifespan by 14.27 % in the FEM-1 (HC17) strain. Interestingly, under lethal oxidative stress, mean life span was increased specifically by 65.05%[90]. Unlike the study by Spindler et al. 2013, mice experienced stress, and suggested an important role for EGCG's anti-oxidant properties to extend life span, possibly via the up-regulation of endogenous antioxidant enzymes, reducing inflammation, which could be useful as an inflammatory protectant against ingestion of excessive air-pollution. It may be that inclusion in food versus beverage may have a different effect.

These external stresses may include dietary factors such as fat intake, but also air-pollution induced adipogenesis, and the formation of inflammatory molecules and enzymes such as superoxide ($O_2^-$), and lipid hydroperoxide (LPO) and the corresponding up-regulation of anti-oxidant enzymes such as SOD, which catalyze the dismutation of $O_2^-$ into $O_2$ and $H_2O_2$. Interestingly, one study showed that intake of dietary fat accelerates the ageing process via increase mortality in *Drosophila melanogaster*. The mechanism is thought to be via accumulating lipid hydroperoxide, when dietary fat was increased from 0–25%, which was partially reversed by green tea catechins in a 5% fat diet, and corresponding lowering of LPO. Inversely, catalase and SOD increased in the GTC or BE diet fed flies[91]. Given the multitude of positive aspects discussed, there may be inherent toxicities of green tea consumption related to its growing environment and need to be mentioned within this review to caution the reader to the consumption of some green tea grown in certain regions of China.

**3.13 Potential Toxicity Concerns of Green Tea Cultivated in Polluted Areas:** This paper has addressed mainly the pure form of EGCG, and not consumed in a tea leaf itself. Concerns regarding sourcing green tea cultivation in China and India, relate not only to air and soil pollution but also the use of harmful pesticides and herbicides. In a report by Harris et al.[92], the authors suggested that the method of consumption of raw Chinese herbal medicines, that nearly 95% possessed levels of heavy metals or pesticides, with most safe for human consumption at levels detected. Further, the residue of hexachlorocyclohexane found in soil and water has been observed to accumulate in the roots, and leaves of teas plants in Fujian province, China[93]. Certainly the majority of registered tea companies are mandated by the government to conduct regular testing of tea products to ensure highest quality and they meet certain national and international food standards. However, the origin of the tea and food safety GMP must be considered before considering inherent pollutants in the tea that may be present due to environmental pollution during tea growth and post-harvest processing.

## 4 Conclusions

This article reviewed the role of green tea not only in traditional DIO, but also discussed its therapeutic use as a depurative and anti-oxidant in air-pollution exposure, in particular diesel exhaust induced obesity in both those exposed and their progeny. EGCG in it pure form and also in its traditional form in green tea with caffeine or novel green tea functional foods represent an effective and culturally acceptable method in China to deliver a daily dietary treatment of both DIO, type 2 diabetes and also the ever increasing pandemic of air-pollution induced obesity (APIO). APIO is a new and growing threat to China's public health from a respiratory, body composition and systemic inflammation perspective. The mechanisms of EGCG dietary modulation is multi-factorial but also presents an interesting target for the inhibition of air-pollution induced obesity. Further research needs to be conducted into the role of tea but also other herbal medicines currently un-investigated for their anti-inflammatory and depurative roles in both ameliorating the inflammatory effects of air-pollution and also the associated obesity protective effects.

**Open Access** This article is distributed under the terms of the Creative Commons Attribution License which permits any use, distribution, and reproduction in any medium, provided the original author(s) and source are credited.
